# Administration of Triclabendazole Is Safe and Effective in Controlling Fascioliasis in an Endemic Community of the Bolivian Altiplano

**DOI:** 10.1371/journal.pntd.0001720

**Published:** 2012-08-07

**Authors:** Fidel Villegas, René Angles, René Barrientos, Gary Barrios, María Adela Valero, Kamal Hamed, Heiner Grueninger, Steven K. Ault, Antonio Montresor, Dirk Engels, Santiago Mas-Coma, Albis Francesco Gabrielli

**Affiliations:** 1 Veterinary Public Health, Pan American Health Organization/World Health Organization, La Paz, Bolivia; 2 Faculty of Medicine, Universidad Mayor de San Andrés, La Paz, Bolivia; 3 Servicio Departamental de Salud/SEDES – La Paz, La Paz, Bolivia; 4 Programa Nacional de Zoonosis – Ministerio de Salud y Deportes, La Paz, Bolivia; 5 Parasitology Department, Faculty of Pharmacy, Universidad de Valencia, Valencia, Spain; 6 Tropical Medicines, Novartis Pharmaceuticals Corporation, East Hanover, New Jersey, United States of America; 7 Tropical Medicines, Novartis Pharma AG, Basel, Switzerland; 8 Regional Program on Neglected Infectious Diseases, Pan American Health Organization/World Health Organization, Washington, D.C., United States of America; 9 Neglected Tropical Diseases, World Health Organization, Geneva, Switzerland; Swiss Tropical and Public Health Institute, Switzerland

## Abstract

**Background:**

The Bolivian northern Altiplano is characterized by a high prevalence of *Fasciola hepatica* infection. In order to assess the feasibility, safety and efficacy of large-scale administration of triclabendazole as an appropriate public health measure to control morbidity associated with fascioliasis, a pilot intervention was implemented in 2008.

**Materials and Methods:**

Schoolchildren from an endemic community were screened for fascioliasis and treated with a single administration of triclabendazole (10 mg/kg). Interviews to assess the occurrence of adverse events were conducted on treatment day, one week later, and one month after treatment. Further parasitological screenings were performed three months after treatment and again two months later (following a further treatment) in order to evaluate the efficacy of the intervention.

**Results:**

Ninety infected children were administered triclabendazole. Adverse events were infrequent and mild. No serious adverse events were reported. Observed cure rates were 77.8% after one treatment and 97.8% after two treatments, while egg reduction rates ranged between 74% and 90.3% after one treatment, and between 84.2% and 99.9% after two treatments. The proportion of high-intensity infections (≥400 epg) decreased from 7.8% to 1.1% after one treatment and to 0% after two treatments.

**Conclusion:**

Administration of triclabendazole is a feasible, safe and efficacious public health intervention in an endemic community in the Bolivian Altiplano, suggesting that preventive chemotherapy can be applied to control of fascioliasis. Further investigations are needed to define the most appropriate frequency of treatment.

## Author Summary

Fascioliasis is highly prevalent in the northern Altiplano of Bolivia. We wanted to ascertain whether a preventive chemotherapy approach, involving the large-scale distribution of triclabendazole within endemic communities, would be feasible for controlling morbidity associated with this disease. Consequently, we implemented a pilot intervention among schoolchildren in a community near Lake Titicaca and assessed this intervention's safety (by evaluating the occurrence of adverse events following treatment) and its efficacy (by measuring changes in prevalence and intensity of infection). Adverse events on treatment day, and one week and one month later were infrequent and mild, and no serious adverse events were reported. We observed cure rates of 77.8% after one treatment and 97.8% after two treatments, egg reduction rates of 74–90.3% after one treatment and 84.2–99.9% after two treatments, and a decrease in the proportion of high-intensity infections (≥400 epg) from 7.8% to 1.1% after one treatment and to 0% after two treatments. We conclude that administration of triclabendazole is a safe and efficacious public health intervention for control of fascioliasis in an endemic community in the Bolivian Altiplano. Preventive chemotherapy with triclabendazole, without individual-level diagnosis and treatment, appears therefore as a feasible option. However, further investigation is needed to define the most appropriate frequency of treatment.

## Introduction

Preventive chemotherapy, the large-scale administration of anthelminthic drugs to population groups at risk, is recommended by WHO for control and elimination of lymphatic filariasis, onchocerciasis, schistosomiasis and soil-transmitted helminth infections [1]. The aim of preventive chemotherapy is to regularly reduce worm load in infected individuals, thus controlling the associated morbidity and decreasing transmission rates. Biological and epidemiological similarities between *Fasciola* spp. and the helminths responsible for the diseases mentioned above, suggest that morbidity associated with fascioliasis could also be controlled through preventive chemotherapy by keeping intensity of infection at low levels among populations at risk [2].

Fascioliasis is a snail-borne zoonosis that can be transmitted to humans through the consumption of raw aquatic vegetables or fresh water contaminated with the cystic larval stages of the worms (metacercariae) [3]. Recent, conservative estimates on the burden of fascioliasis indicate that the number of individuals infected worldwide is at least 2.65 million, and more than 50% of them live in Latin America [4].

*F. hepatica* is the only liver fluke species transmitted in Bolivia [5], where endemic communities face among the highest prevalence and intensity of *F. hepatica* infection in the world [6]–[9]. The area endemic for *talp'a laqu*, as fascioliasis is known in the local Aymara language, is limited to a relatively small region (60×60 km) of the northern Altiplano (i.e. the plain between Lake Titicaca and the capital city La Paz) [8], where transmission is linked to the presence of rivers and subsoil effluences inhabited by the intermediate snail host, *Galba truncatula*. In this region, the main reservoirs of infection are domestic animals, including ovines, bovines, porcines and equines [10].

In humans, fascioliasis is associated with an acute clinical phase resulting from the migration of the immature worms through the liver. Symptoms include fever, abdominal pain, respiratory disturbances and skin rashes. The chronic phase starts when the worms reach the bile ducts: progressive inflammation leads to fibrosis and thickening of the walls of the biliary system and of the surrounding hepatic tissue. Biliary colic pain due to blockage of the bile ducts and jaundice are possible complications. Severe infections may result in biliary cirrhosis with scarring and fibrosis of the liver [3]. Anaemia is a common finding in both acute and chronic fascioliasis [11]–[14].

Triclabendazole is the WHO-recommended essential medicine for treatment of fascioliasis [15]. The range of the cure rate produced by a single 10 mg/kg administration is 78–100% [16]–[21], while information on egg reduction rate (ERR) is less abundant: three studies conducted in Egypt reported ERR of 73% and 100% based on arithmetic means [18], [19], and 63% based on geometric means [21]. Triclabendazole is generally regarded as a safe drug, although adverse events (AEs) can occur following treatment [16], [17]. Such events are directly proportionate to intensity of infection and can be classified as systemic or mechanical. Systemic AEs are caused by biological substances released by the dying worms and include mild/transient dizziness, headache, nausea, and urticaria. Mechanical events are generally linked to the expulsion of dead worms from the biliary system towards the intestinal lumen, and include biliary colic pain, possibly associated with jaundice.

Treatment with triclabendazole has usually been implemented in a clinical setting while its use in public health interventions is limited. In Egypt, however, a triclabendazole treatment programme based on selective chemotherapy (test-and-treat) of school-age children has been implemented in six districts in the Nile Delta area since 1998 [22], [23], while in Vietnam treatment has been decentralized since 2006 and is administered in peripheral hospitals and health posts based on a simplified diagnostic protocol [AF Gabrielli, personal communication]. In Bolivia, where prevalence of infection is higher than in Egypt or Vietnam, a more inclusive strategy offering treatment to entire population sectors without individual diagnosis might be appropriate in order to reduce costs and logistics related to the implementation of screening exercises. This approach would mirror the one currently recommended for schistosomiasis and soil-transmitted helminthiasis in areas of moderate and high risk [1]; the procurement of larger quantities of medicines needed for its implementation has been made possible via the donation of triclabendazole (Egaten) by Novartis Pharma AG through the WHO.

Consequently, in 2008, following suggestions by a panel of experts convened by the WHO [24], the Ministerio de Salud y Deportes of Bolivia decided to opt for large-scale distribution of triclabendazole in endemic areas without individual diagnosis. Before this approach was widely implemented, a pilot study was conducted to test the safety and efficacy of such intervention. Safety assessments consisted of monitoring and recording any AEs occurring after treatment, and efficacy was assessed by monitoring prevalence and intensity of infection and by calculating cure and egg reduction rates. Safety, in particular, was considered as a key component of the protocol as AEs are known to limit the feasibility of preventive chemotherapy interventions for helminth infections, as their occurrence confines treatment to a clinical setting where proper management of cases is ensured by health personnel. AEs also have the potential to jeopardize compliance to the intervention as effects of treatment can be perceived as a greater health risk than the disease itself [25].

## Methods

### Ethics

The study protocol was approved by the Comisión de Etica de la Investigación (CEI) of the National Bioethics Committee of Bolivia on September 10, 2007. A written informed consent form explaining the purpose and the modalities of the study was developed, translated into Aymara and obtained from the parents/guardians of each participating child. The initiative was agreed by the civil (sub-alcalde, head of the health post, director of the educational unit), and traditional (jilakatas and malkus) authorities of the community where the study was implemented.

### Study setting

The study was conducted in Huacullani, a community in the Bolivian northern Altiplano, where prevalence of *F. hepatica* infection among school-aged children ranged between 31.2% and 38.2% in the 1990s [9]. Huacullani (16°26′0″S, 68°44′0″W) is located at an altitude of 3,850 metres, approximately 500 meters from the shores of Lake Titicaca, in the municipality of Tihuanaku (province of Ingavi, department of La Paz). At the time of the survey, the population of Huacullani was 2,472.

### Study population

School-aged children (5–14 years) were selected as the target group of the intervention, as they are at higher risk of infection and morbidity. Children are more likely than adults to become infected, as exemplified by their higher levels of prevalence of infection, and to develop mechanical AEs following treatment, because of the smaller size of their bile ducts and thus higher likelihood of blockage. Consequently, they are considered both the group at highest risk and the one most sensitive for detection of AEs following treatment. All children attending the primary school and the junior high school of Huacullani were considered eligible for enrolment in the study.

### Study design

**Figure 1:**
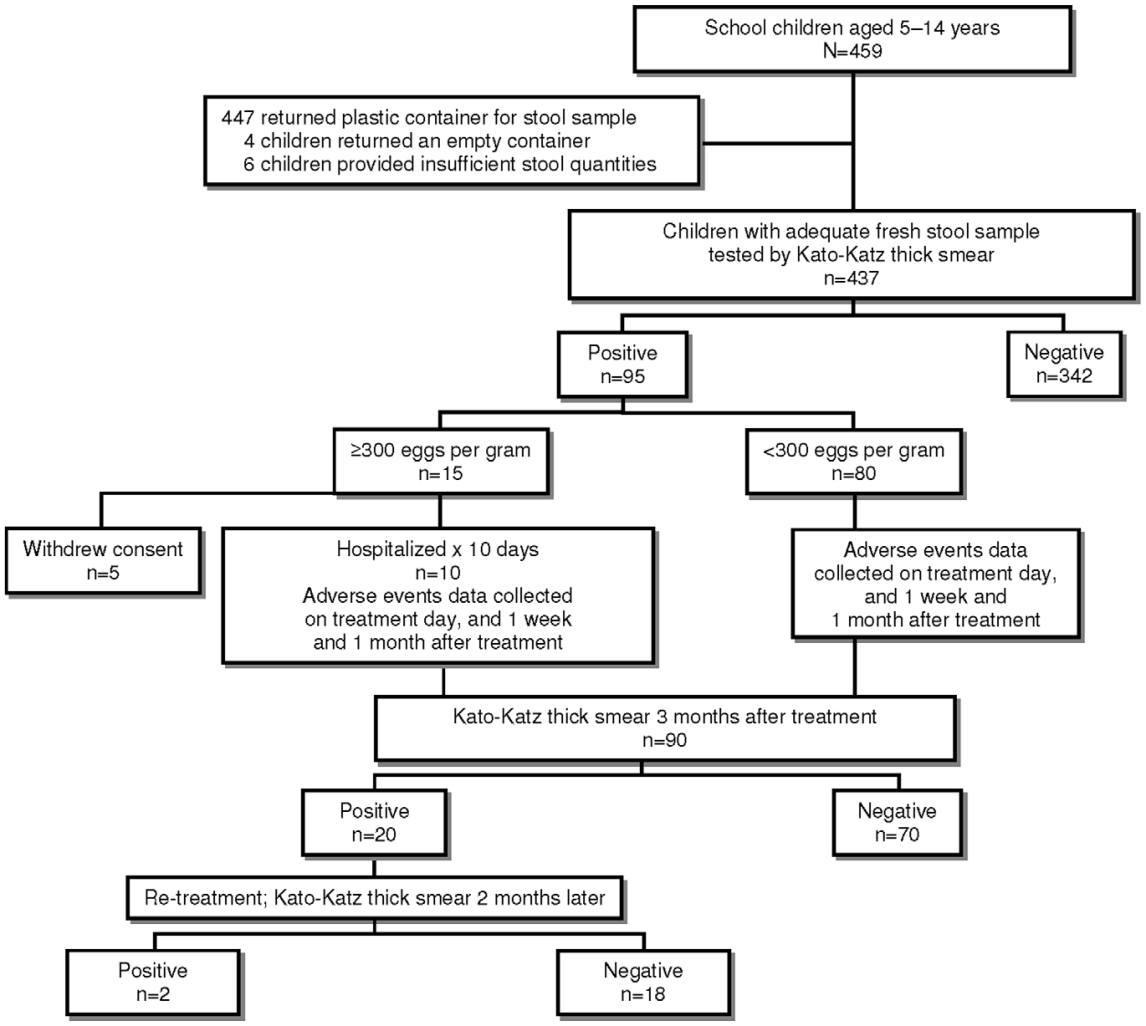
Study flow diagram.

A Scientific Committee formed by the Ministerio de Salud y Deportes, the Servicio Departamental de Salud of La Paz, the Universidad Mayor de San Andrés and the PAHO/WHO was established with the aim of developing a protocol and supervising the implementation of the pilot intervention. The protocol consisted of five consecutive study phases: baseline data collection; treatment; monitoring of AEs at day 0, day 7 and day 30; first parasitological follow-up 3 months after treatment, with further treatment of any cases still positive; and second parasitological follow-up 2 months after the first follow-up (Figure 1). After a few preparatory meetings, field activities started in April 2008, and were completed in November 2008.

#### Baseline data collection

Each participating child was given a plastic numbered container on the day of the survey and was asked to return it with a fresh stool sample. A single Kato-Katz thick smear [26], [27] was prepared from each stool sample. The Kato-Katz test was chosen as – in spite of its recognized low sensitivity – it is the standard technique used for assessing burden of helminth infections at community level [1], and also allows a quantitative assessment of the severity (intensity) of the infection, through a measurement of egg density in the faecal sample. Each individual was therefore classified as egg-positive or negative, and intensity of infection (number of eggs per gram of faeces, epg) was assessed in all positive individuals. Prevalence of infection and mean intensity of infection were thus calculated.

#### Treatment

All children who tested positive to the Kato-Katz technique were considered eligible for treatment. Triclabendazole (Egaten, Novartis Pharma AG, Basel, Switzerland) was administered at an approximate dose of 10 mg/kg, in a single administration, based on the schema presented in Table 1. Children with an intensity of infection ≥300 epg were hospitalized at the Ovidio Aliaga Uría Children's Hospital in La Paz before receiving treatment. This precautionary measure was justified by the consideration that individuals infected with a large number of worms are more likely to develop both systemic and mechanical AEs following treatment and might therefore require specialist medical attention and care. The 300-epg threshold was adapted from the 400-epg threshold used for identifying high-intensity infections with *Schistosoma mansoni* [28], which was subsequently adopted for *Fasciola* spp. infections as well [29]. In the context of the pilot intervention, the 400-epg threshold was further lowered by 100 units as an additional precautionary measure. All other children were treated on an outpatient basis, at school premises, under the supervision of their teachers and medical staff. In addition, the local health post at Huacullani was alerted and ready to provide medical care in case of need.

**Table 1:** Weight-based dosing of triclabendazole and number of tablets administered.

Weight (kg)	Dose (mg)	Number of triclabendazole tablets
≤12.5	125	½
>12.5 and ≤25	250	1
>25 and ≤37.5	375	1 ½
>37.5 and ≤50	500	2
>50 and ≤62.5	625	2 ½
>62.5 and ≤75	750	3
>75 and ≤87.5	875	3 ½
>87.5 and ≤100	1000	4

#### Monitoring of adverse events

Children treated at school were kept under observation for approximately three hours after drug administration, after which they were interviewed following a structured protocol aimed at detecting any AE that may have occurred in the meanwhile. Axillary body temperature was also measured by electronic thermometer. Detected events were classified as “adverse events” (AEs) or “serious adverse events” (SAEs). SAEs were defined as events that are fatal, life-threatening, disabling, incapacitating or that cause or prolong hospitalization after drug intake [1], while any less serious event was classified as an AE. Such children were also followed up on the school premises one week and one month after treatment, and interviewed again. Children treated at the hospital were kept under observation at the hospital premises for 10 days, and interviewed on treatment day and after one week; they were also interviewed again one month after treatment at school, together with the other children.

#### Parasitological follow-ups

Three months after administration of triclabendazole, the efficacy of treatment among all treated children who had tested positive at baseline was assessed by another single Kato-Katz thick smear. All children testing positive at this first parasitological follow-up were treated again (triclabendazole, 10 mg/kg, single administration) and reassessed two months later (second parasitological follow-up), with the same technique as the first follow-up. Based on measured prevalence and arithmetic mean intensity of infection, cure and egg reduction rates were calculated for the population under study at both follow-ups. For logistic reasons, only positive cases were progressively followed up: children negative at baseline were therefore not included in the first follow-up, and children negative at first follow-up were not included in the second follow-up.

#### Data management and analysis

Data were collected and analysed by staff of the Ministerio de Salud y Deportes, the Servicio Departamental de Salud of La Paz, the Universidad Mayor de San Andrés and the PAHO/WHO who directly supervised and participated in the activities described in this paper.

## Results

### Baseline data collection

At the time of the baseline survey (April 2008), the school population of Huacullani consisted of 459 children aged 5 to 14 years, who were all considered eligible for treatment. 447 children returned the plastic container. In total, 437 faecal samples from an equivalent number of children were examined by the Kato-Katz thick smear technique – 4 children returned an empty plastic container, and 6 other children provided insufficient stool quantities to prepare a Kato-Katz slide. Stool samples were transported to the Faculty of Medicine of the Universidad Mayor de San Andrés in La Paz and processed. Slides were read within 24 hours of preparation.

Overall, 95 children had positive and 342 had negative Kato-Katz smears. The parasitological prevalence of *F. hepatica* infection in this population was therefore 21.7%. Among the 95 children with positive Kato-Katz smears, 15 had an intensity of infection ≥300 epg (15.8%), and 11 a high-intensity infection (≥400 epg, 11.6%). The mean intensity of infection among all surveyed children (including the ones with negative smears) was 72.9 epg.

### Treatment

Triclabendazole was administered in June 2008 to each child testing positive to the Kato-Katz test. Among the 15 children with an intensity of infection ≥300 epg, 10 were hospitalized before treatment, while 5 could not be treated as their parents refused hospitalization and/or treatment. By contrast, all the 80 Kato-Katz positive children with an intensity of infection <300 epg were treated as outpatients at school premises. In total, 90 children were administered triclabendazole: among them, the mean intensity of infection was 264.3 epg, and 7 had a high-intensity infection (≥400 epg, 7.8%).

### Adverse events following treatment

Among the 90 treated children, the number reporting one or more AEs on treatment day and one week after treatment (June 2008) was 11 and 10, respectively. One month after treatment (July 2008), only 82 children were interviewed, as 8 were neither at school nor could be traced in Huacullani; among them, only three children reported any AE. Details are provided in Table 2.

**Table 2:** Adverse events experienced by children treated with triclabendazole.

Adverse event	Day 0^a^	Day 7^b^	Day 30^c^
Abdominal pain	2	4	2
Fever	–	1	–
Nausea	1	–	–
Fatigue	–	1	–
Headache	5	2	1
Abdominal pain and nausea	1	–	–
Abdominal pain and headache	–	1	–
Headache and fever	1	–	–
Abdominal pain, headache and fever	1	–	–
Headache, fatigue and fever	–	1	–
**Number of AEs reported or observed**	15	13	3
**Number of children with AEs**	11	10	3
**Proportion of children with AEs among those observed**	12.2%	11.1%	3.7%

a n = 90 children;

b n = 90 children;

c n = 82 children

The number of reported AEs on treatment day, one week after treatment and one month after treatment was 15, 13 and 3, respectively. Headache was the most frequent event reported on treatment day, and abdominal pain was the most frequent one week later. All fevers were below 38°C. Only 3 of the children experiencing AEs on treatment day also reported an AE one week after treatment. Only 1 of the children with a high-intensity infection (≥400 epg) reported an AE one week after treatment (abdominal pain).

Among children treated at school, only one girl requested to be taken to the local health post on treatment day, but after a medical examination, she did not require any specific medical attention, and all the signs and symptoms resolved spontaneously. None of the other children contacted the health post for medical assistance during the follow-up period.

Overall, no medications were administered to treat AEs with the exception of antipyretics in case of fever. No SAEs occurred.

### Efficacy of treatment

#### First parasitological follow-up

The first parasitological follow-up was carried out approximately three months after treatment (September 2008). Collection and processing of faecal samples followed the same procedures as baseline data collection.

Among the 90 treated children, 20 were still positive by Kato-Katz smear at the first parasitological follow-up (22.2%); this is equivalent to a parasitological cure rate of 77.8%. Among the 20 positive children, only 1 had a high intensity (≥400 epg). Table 3 summarizes this information.

**Table 3:** Chronological evolution of the number of positive cases and the cure rate.

	Negative	Positive		Cure rate
		n<400 epg	n≥400 epg	
**Baseline (n = 437)**	342	84 (83)	11 (7)	–
**1^st^ follow-up (n = 90)**	70	19	1	77.8%
**2^nd^ follow-up (n = 20)**	18	2	0	97.8%

Note: figures in brackets refer to children who were treated (n = 90).

At first parasitological follow-up, the mean intensity of infection among all those sampled at baseline was 7 epg, and among the whole treated population 33.9 epg. The respective ERRs from baseline levels were 90.3% and 87.2%. The mean intensity of infection among the 20 positive cases alone was 152.4 epg. Table 4 summarizes this information.

**Table 4:** Chronological evolution of intensity of infection and egg reduction rates (ERRs).

Time of observation	Among those sampled at baseline (n = 437)		Among those treated at baseline (n = 90)		Among those positive at 1^st^ follow-up (n = 20)	
	Mean epg	ERR	Mean epg	ERR	Mean epg	ERR
**Baseline**	72.9	–	264.3	–	585.4	–
**1^st^ follow-up**	7*	90.3%	33.9	87.2%	152.4	74%
**2^nd^ follow-up**	0.1*	99.9%	0.5	99.8%	24	84.2%

* As children negative at baseline were not followed up, calculations are based on the assumption that all of them remained negative at 1^st^ and 2^nd^ follow-up.

Following completion of the first parasitological follow-up, the 20 children still classified positive by Kato-Katz smear were treated again following the schema presented in Table 1.

#### Second parasitological follow-up

Collection and processing of samples at the second parasitological follow-up (November 2008) also followed the procedure for baseline data collection. After the second dose of triclabendazole, only two children were still positive by the Kato-Katz test: parasitological cure rate from baseline was therefore equivalent to 88/90 (97.8%) (Table 3). Mean intensity of infection for the two positive cases at second parasitological follow-up was 24 epg, while that among all those followed up was 0.5 epg. ERR between baseline an 2^nd^ follow-up was 84.2% when calculated only among those still positive at 1st follow-up, 99.8% among those positive and treated at baseline, and 99.9% among all those sampled at baseline (Table 4).

## Discussion

Overall, 21.7% of the children surveyed were found to be infected with *F. hepatica* at baseline. This was less than the prevalence of infection previously detected in Huacullani [9], but was nevertheless high when compared with the usually low levels of *F. hepatica* in most endemic countries across the world, such as Egypt, Iran, Vietnam or Yemen for example, where prevalence of infection by faecal examination rarely exceeds 5% [10], [22], [23], [30]–[32]. It is also likely that the true prevalence of infection is higher due to the low sensitivity of a single Kato-Katz smear.

Treatment with a single administration of triclabendazole (10 mg/kg) did not elicit frequent or considerable AEs, neither among children with a high intensity of infection, nor among the others. No significant medical attention was required in any case, as all symptoms resolved spontaneously without any appreciable consequence on the health status of treated individuals. The occurrence of AEs documented by our study contrasts with the absence of any event reported in Egypt [23] even though both the treatment regimen and the manufacturer of triclabendazole were the same. While comparison might not be fully appropriate, as in Egypt no active search of events was carried out, such discrepancy might be attributable to the lower mean intensity of infection observed in this country (12.2 epg at baseline). Frequency and severity of AEs are however expected to be less important at subsequent rounds of treatment in reason of the progressively decreasing intensity of infection, as shown by experiences from different helminth control interventions implemented across the world [1], [21], [33].

The parasitological cure rate achieved after a single administration of triclabendazole at 10 mg/kg was high (77.8%) and consistent with previous reports in the scientific literature for this treatment course [27]–[29]. ERRs were also considerable, even though lower rates were observed among individuals with a higher intensity of infection at baseline (Table 4). The negative relationship between ERR and baseline intensity of infection has been described in the case of other helminth infections, such as those by *Trichuris trichiura*: both density-dependent fecundity and reduced bioavailability of triclabendazole per adult worm have been proposed as possible explanatory hypotheses [34], [35]. Finally, only 1.1% of the 90 treated children still had high-intensity infections (≥400 epg) at the first parasitological follow-up, compared to 7.8% at baseline. If we apply to fascioliasis the model described in other helminth infections, that intensity of infection is proportionate to morbidity [1], [2], it can be inferred that morbidity was under control in a very high proportion of children three months after a single administration of triclabendazole 10 mg/kg.

Based on the results of the pilot intervention, we conclude that triclabendazole is a safe and efficacious drug when administered to a paediatric population living in a fascioliasis endemic area. These considerations suggest that a population-based drug distribution approach, without individual diagnosis and without direct medical supervision, in a manner comparable with the preventive chemotherapy interventions implemented worldwide against the four major helminth infections, is appropriate and feasible.

Notably, triclabendazole was well tolerated across the population examined, including individuals with a high intensity of infection: AEs elicited were self-limiting, did not require any specialist medical attention and could be managed by the local health staff. In terms of efficacy, a single administration of triclabendazole was effective in reducing considerably the number of infected individuals, the mean intensity of infection and the proportion of high-intensity infections, and in keeping these indicators at low levels for a few months after treatment.

Surveys with a longer follow-up are recommended in order to ascertain for how long a single administration of triclabendazole can sustain low prevalence and intensity of infection in endemic areas. Such a study would allow the most appropriate interval of re-treatment to be determined.

Following the successful implementation of the pilot intervention, the health authorities of Bolivia decided to implement distribution of triclabendazole on a large scale.
